# Supplementary material for: Direct observation of dynamic modes excited in a magnetic insulator by pure spin current
Source: Sci Rep. 2016 Sep 9;6:32781. doi: 10.1038/srep32781 (PMC5016739; doi:10.1038/srep32781)
Supplement: Supplementary Information [file srep32781-s1.pdf]

Direct observation of dynamic modes excited in a magnetic insulator by pure spin current

V. E. Demidov, M. Evelt, V. Bessonov, S. O. Demokritov, J. L. Prieto, M. Muñoz, J. Ben Youssef, V. V. Naletov, G. de Loubens, O. Klein, M. Collet, P. Bortolotti, V. Cros and A. Anane

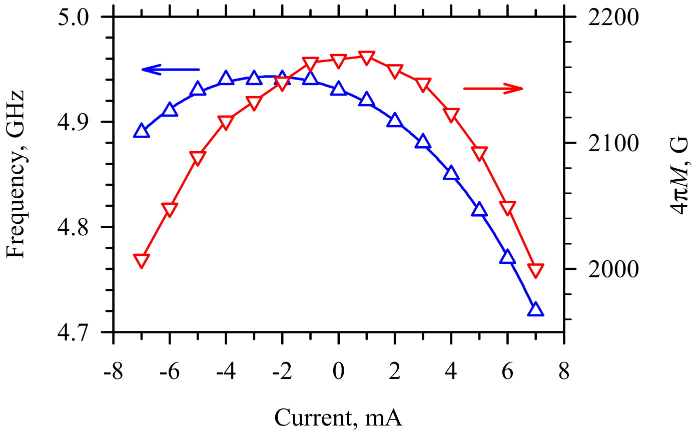

Supplementary Figure S1. BLS measurements of the ferromagnetic resonance in the YIG disk. In order to measure the ferromagnetic resonance (FMR) in the YIG disk, we transmit a microwave current through an additional 5 μm wide stripe antenna, which creates a nearly uniform in-plane dynamic magnetic field across the YIG disk. By varying the frequency of the excitation signal in the antenna and measuring the BLS intensity, we record the FMR curves and obtain the FMR frequency. Additionally, the electrical dc current $I<I_C$ is applied through the Pt layer to analyze its influence on the FMR. The FMR frequency obtained for different currents is shown by up-triangles. The observed variation of the frequency originates from the Oersted field of the current and the reduction of the static magnetization M caused by the Joule heating of the sample. We estimate the Oersted field from the Ampere's law and calculate the current

dependence of the magnetization by using the Kittel formula for the FMR frequency. The results shown by the down-triangles indicate that M is reduced by about 8% at I=7 mA. The obtained dependence is nearly symmetrical with respect to the change of the sign of the current, in agreement with the expectations for the effects of the Joule heating. The data were obtained at H_0=1000 Oe. Lines are guides for the eye.
